# SLC6A4 gene variants moderate associations between childhood food insecurity and adolescent mental health

**DOI:** 10.1002/brb3.3426

**Published:** 2024-02-15

**Authors:** S. Pilkay, M. Nolasco, S. Nunes, A. Riffer, D. Femia, D. Halevy, T. Veerman, S. Heiland, N. Suwannimit, N. Trexler, B. Gump

**Affiliations:** ^1^ David B. Falk College of Sport and Human Dynamics, School of Social Work Syracuse University Syracuse New York USA; ^2^ Jane Addams College of Social Work University of Illinois Chicago Chicago Illinois USA; ^3^ Wurzweiler School of Social Work Yeshiva University New York New York USA; ^4^ School of Natural Health, Social and Behavioral Sciences, Social Work Centenary University Hackettstown New Jersey USA; ^5^ David B. Falk College of Sport and Human Dynamics, Department of Public Health Syracuse University Syracuse New York USA

**Keywords:** adolescence, anxiety, food insecurity, gene–environment, SLC6A4

## Abstract

**Background:**

Food insecurity is a persistent concern in the United States and has been shown to affect child mental health and behavior. The SLC6A4 gene has been indicated as a moderator of the effects of chronic stress on anxiety in adolescents aged 14–21. However, it is unclear if SLC6A4 may also play a role in the effects of childhood food insecurity, a form of chronic stress, on adolescent mental health. This study aimed to identify effects of food insecurity on adolescents’ mental health and delinquent behavior when both mom and child go hungry in the child's early years, and the potential interaction with SLC6A4 variants (SS/LL).

**Methods:**

The data and sample for this research are from the Future of Families and Child Wellbeing Study. The cohort consists of 4898 children (age 1–15 years, male = 47%, African American = 50%) and their respective caregivers sampled from large cities in the United States from 1998 to 2000.

**Results:**

The SLC6A4 serotonin transporter short/short allele emerged statistically significant as a moderator of childhood food insecurity and adolescent mental health. Specifically, the presence of the short/short allele increased anxiety symptoms in adolescents with exposure to food insecurity in childhood.

**Conclusion:**

The SLC6A4 short/short allele amplifies risk of anxiety‐related mental illness when children experience food insecurity. The gene–environment interaction provides insight into the mechanistic pathway of the effects of poverty‐related adversity, such as food insecurity, on developmental trajectories of mental health.

## Graphical abstract

Food insecurity is a persistent concern in the United States and has been shown to affect child mental health and behavior. The SLC6A4 gene has been indicated as a moderator of the effects of chronic stress on anxiety in adolescents aged 14–21. This study aimed to identify the effects of food insecurity on adolescents' mental health and delinquent behavior when both mom and child go hungry in the child's early years, and the potential interaction with SLC6A4 variants (SS/LL).

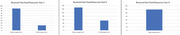

## 1 INTRODUCTION

The issue of food insecurity remains a prevalent and ongoing concern within the United States. Extensive research conducted in the field of mental and behavioral health, specifically by Edmunds and Alcaraz (2021), has demonstrated the adverse impact of food insecurity on the development and overall well‐being of children. According to the United States Department of Agriculture (USDA), food insecurity is characterized as the inability to obtain sufficient food as a result of financial constraints or other insufficiencies in resources (USDA, 2020). Food insecurity is a comprehensive concept that encompasses the inability to lead a healthy lifestyle and experiencing dissatisfaction with regards to the quantity and diversity of one's food (Schroeder & Smaldone, 2015). Based on the Food Security Survey conducted by the United States Department of Agriculture (USDA) in 2020, it is apparent that there is a notable presence of food insecurity within households in the United States. This is supported by the fact that 10.5% or approximately 13.1 million US households reported experiencing food insecurity at various times throughout the year 2020, as documented by the USDA (2020).

Food insecurity is assessed using two distinct categories: low food security and very low food security. Low food security is distinguished by a decline in the overall quality of one's diet and a limited range of dietary options. Food insecurity has also been assessed using indicators such as individuals' level of contentment with their food intake and their confidence in the consistent availability of food (Schroeder & Smaldone, 2015). According to the United States Department of Agriculture (USDA, 2020), individuals experiencing very low food security exhibit irregular meal patterns and insufficient food consumption as a result of their household's inability to get an adequate food supply. Furthermore, it was observed that homes comprising children had a higher propensity for experiencing food insecurity compared to households without children, with prevalence rates of 14.8% and 8.8% respectively (USDA, 2020). According to the United States Department of Agriculture (USDA) in 2020, out of the total 13.1 million families examined, approximately 6.1 million households were found to be food insecure, with an additional 580,000 households categorized as experiencing low food security. In the wake of the worldwide COVID‐19 pandemic, there has been a notable threefold increase in complaints of food insecurity among households with children (Schanzenbach & Pitts, 2020).

Research has demonstrated that food insecurity exerts a significant impact on the mental well‐being and behavioral patterns of children. According to a study conducted by Men et al. (2021), there is a higher likelihood of Canadian kids residing in food‐insecure homes to report mood disorders, anxiety disorders, and emotional discomfort as compared to those living in food‐secure households. Moreover, there is a significantly elevated risk of suicidal ideation among children who experience food hardship, with the likelihood being up to 6.49 times greater (Men et al., 2021). In their study, Edmunds and Alcaraz (2021) observed a correlation between childhood material hardship, specifically during the ages of 9 and/or 15, and the manifestation of anxiety and depressive symptoms in adolescence at the age of 15. Edmunds and Alcaraz (2021) assessed material hardship through the absence of essential necessities, with adequate food within the household being considered a fundamental requirement. This measurement encompassed several poverty‐related characteristics associated with material hardship. The presence of food insecurity among children and adolescents is associated with an increased likelihood of experiencing behavior problems (Greder et al., 2017), internalizing these behavior problems (Burke et al., 2016), and a heightened risk of engaging in alcohol and substance use (Turner et al., 2022). Children experience a range of developmental milestones from birth until the age of 5. These milestones can be affected by stresses associated with food insecurity, both alone and in combination with other stressors (CDC, 2022).

Several studies have demonstrated the involvement of the serotonin transporter gene SLC6A4 in gene X environment interactions across several developmental outcomes in both humans and animals. For instance, Wilson and Kinkead (2008) demonstrated that the SLC6A4 gene has a role in regulating the negative consequences of social subordination on the timing of puberty start in rhesus monkeys. Specifically, individuals carrying the short form of the gene exhibited delayed sexual development when subjected to subordination. According to a study conducted by Brody et al. (2009), the presence of the SLC6A4 long/long allele was found to decrease the likelihood of engaging in risky behavior among African American youths (approximately 11.2 years old) who were participating in the Strong African American Families prevention program. This effect was observed when comparing these individuals to their peers who did not possess the allele and were also enrolled in the program, as well as to peers who both did and did not possess the allele but were not enrolled in the program. Furthermore, a study conducted by Uher et al. (2011) found that individuals who possessed two short alleles of the SLC6A4 gene and had a history of childhood abuse exhibited a heightened susceptibility to enduring depression as compared to those who did not possess the two short alleles.

The potential influence of gene–environment interactions on the impact of food insecurity on infant development can be mediated by stress. The study conducted by Ollmann et al. (2021) discovered that the SLC6A4 gene plays a moderating role in the relationship between chronic stress and anxiety among individuals in the age range of 14–21 years. Multiple studies have demonstrated the interaction between the SLC6A4 gene and stress‐inducing situations, leading to an elevated susceptibility to mental and behavioral health disorders (Ollmann et al., 2021). According to Mueller et al. (2012), the hypothesis posits that specific variations of SLC6A4 have an impact on an individual's physiological reaction to psychological stress. This idea holds significance in the investigation of the consequences of food insecurity on children and adolescents. The primary objective of the current investigation was to ascertain if the SLC6A4 gene plays a moderating role in the relationships between food insecurity, mental health, and delinquent conduct among individuals in the developmental stage of childhood and adolescence.

## 2 METHODS

### 2.1 Sample

The data and sample for this research are from the Future of Families and Child Wellbeing Study. The cohort consists of 4898 children and their respective caregivers sampled from large cities in the United States from 1998 to 2000. Initial data were collected from the mothers shortly after the birth of the child and follow‐up data were collected when the children were of subsequent ages (1, 3, 5, 9, 15, and 22 years). The 1998−2000 baseline data collection comprised mother and father core interviews upon the birth of the study's “focal child.” Hospital interviews followed the child's birth. At baseline and the next five waves, core phone interviews collected data on parental relationships, parenting, health and health behaviors, family and social support, demography, housing, social program use, education, and employment. The 1999−2001 Year 1 follow‐up wave includes mother and father core interviews around the focal child's first birthday. Year 3 (2001–2003) and year 5 (2003–2006) follow‐up waves included mother and father core interviews, primary caregiver interviews, and home visits around the target child's third and fifth birthdays. Primary caregiver interviews addressed health, routines, and parenting. Year 9 (2007–2010) included mother and father core interviews, primary caregiver interviews, house visits, and interviewer observations, as the previous two waves. Around their ninth birthday, the focal child was interviewed regarding family, school, task completion, self‐concept, and home routines. In‐home examinations collected saliva samples from target children and their biological mothers for biomarker data (Telomere Length, DNA Methylation Clocks), genotyping, and Polygenic Scores (PGS). Year 15 follow‐up data were collected 2014−2017. A subgroup of 1000 youths had primary caregiver and teen phone interviews, home visits, and interviewer observations. Phone interviews included focal children's schooling, school experiences, dangerous behaviors like sexual activity and substance use, peer interactions, and prosocial activities.

The child cohort is almost equally divided by sex (Male 47.8%), mostly African American (*n* = 1800) followed by LatinX (*n* = 817), Caucasian (*n* = 780), Asian American (*n* = 102), and Indigenous (*n* = 52) with 27% of the cohort not reporting on race. More than half of the sample were living in households with an annual income below the federal poverty line (68.7%) and the mothers were a mean age of 25.27 years (SD = 6.03) at the time of child's birth.

### 2.2 Variables

The assessment of food insecurity involved the collection of data from mothers regarding instances of missing meals for both themselves and their children. This information was obtained through a subsequent survey that also inquired about financial circumstances and other indicators of economic well‐being and functionality. The questionnaire comprised inquiries that examined comparable phenomena assessed in established food insecurity questionnaires, including concerns about food uncertainty or anxiety (related to the situation, resources, or supply); insufficiency of food (for both adults and children); perceptions of low‐quality food (in terms of dietary diversity, nutritional adequacy, and preference); decreased food consumption (for both adults and children); diminished food intake (for both adults and children); and feelings of embarrassment associated with resorting to socially inappropriate means to obtain food. Nevertheless, due to the absence of a standardized measure for collecting food insecurity data, which could potentially introduce measurement errors and affect the study's results, we made the decision to solely rely on maternal self‐reporting of “missed meals” for both the mother and child at years 1, 3, and 5. The absence of regular meals resulting from insufficient or nonexistent food supplies is considered a key measure of food insecurity. The United States Department of Agriculture (USDA) has defined this condition as “very low food security” (USDA, 2020). By employing this methodology, we were able to assess food insecurity among individuals who were most susceptible to adverse consequences, hence maximizing the potential for observable impacts. The variable “missed meals” was operationalized as the presence or absence of mothers missing meals (coded as 0 for no and 1 for yes) and children missing meals (coded as 0 for no and 1 for yes) at years 1, 3, and 5. The measurement of food resource reception (0 = absence, 1 = presence) was conducted during the first, third, and fifth years. The present study examines the topic of adolescent mental health, specifically focusing on anxiety‐related symptoms as measured by the Brief Symptom Inventory 18. The inventory employs a Likert scale, ranging from 1 (strongly disagree) to 4 (strongly agree), to assess the extent to which participants endorse the following statements: “I experience feelings of nervousness or shakiness internally,” “I experience feelings of fear,” “I feel restless to the point where I am unable to sit still,” “I have episodes of terror or panic,” “I experience feelings of tension or being on edge,” and “I occasionally experience sudden fear without any apparent cause.” The study assessed delinquent conduct among adolescents at the age of 15 using the Delinquent Conduct Scale, which quantifies the frequency of engagement in certain actions on a scale ranging from 1 (never) to 4 (five or more times). The subsequent examples delineate instances of delinquent behavior exhibited by adolescents: “Engaging in the act of shoplifting by taking merchandise without remitting payment,” “Participating in a physically aggressive altercation of significant magnitude,” “Operating a motor vehicle without obtaining the owner's consent,” “Causing harm to an individual to the extent that medical attention or bandages are required,” “Utilizing or employing the threat of force with a weapon to acquire possessions,” “Engaging in the sale of illicit substances such as marijuana or other drugs,” and “Illegally entering a residential or commercial structure with the intent to pilfer belongings.” The variables of the SLC6A4 gene variation encompassed the alleles of short/short and long/long.

### 2.3 Statistical analyses

The examination of cohort demographics was conducted using descriptive statistics. The predictor and outcome variables were evaluated using a correlation matrix in order to discover initial relationships that would be further examined in multiple regression analyses. The multiple regression analyses used potential confounders that were discovered in the correlation matrix. Additionally, bootstrap confidence intervals were employed to mitigate the risk of type I error resulting from spurious associations, potential nonnormality of the data, and the need to account for multiple comparisons. Gene–environment interactions were examined with a computed interaction variable for each of the SLC6A4 variants and the predictor variable of interest controlling for the interaction variables independently and child sex. Subsequently, the statistically significant interactions were graphically shown to provide a visual depiction. Food insecurity was examined, using multinomial logistic regression, for effects on adolescent mental health and delinquent behavior that were indicated as statistically significantly associated with a gene–environment interaction. Food insecurity data for this analysis were coded into a new variable according to timing and chronicity of the food insecurity event for mothers and children (no skipped meals = 0, early exposure year 1 = 1, late exposure year 5 or 9 = 2, chronic exposure having been exposed at an early and late time period = 3). Any statistically significant food insecurity predictors from this analysis were then included as covariates in the gene–environment interaction analyses.

## 3 RESULTS

### 3.1 Food insecurity

**FIGURE 1:**
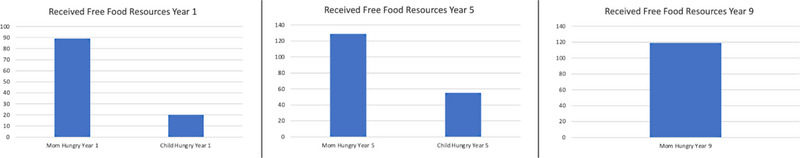
Bar graph of mother and child food insecurity with food resources.

Mothers who reported receiving food resources during the child's first year were more likely to report receiving food resources at year 5 (*r* = .324, *p* = .036), and year 9 (*r* = .534, *p* < .001). However, even with the food resources the mothers still reported skipping meals due to food insecurity during the child's first, fifth, and ninth year of life as shown in Figure 1. Children reported to have gone hungry in year 1 were more likely to be reported going hungry in year 5 (*r* = .468, *p* = .002) and to have their mothers report going hungry in year 5 (*r* = .334, *p* = .031). Maternal reports of mom going hungry in child's first year were more likely to report their child going hungry in year 5 (*r* = .313, *p* = .044). Mothers going hungry during child's fifth year was moderately positively associated with children going hungry during their fifth year (*r* = .465, *p* = .002).

**FIGURE 2:**
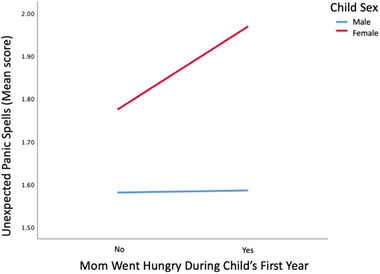
Line graph of maternal food insecurity and child sex moderation of adolescent panic spells.

The initial correlation matrix identified that maternal food insecurity showed a moderate positive association with adolescent anxiety symptoms at year 15. Mothers who reported going without food when hungry during their child's first year had adolescents who reported more unexpected panic spells (*r* = .424, *p* = .005). Moreover, panic spells at age 15 were positively correlated with the adolescent reporting they have used a weapon to threaten a person to steal from them (*r* = .361, *p* = .019). A multiple regression analysis with an interaction term of maternal hunger and child sex as a predictor revealed that child sex moderates the influence of maternal food insecurity on the development of panic spells in adolescence (*B* = 0.299, *t* = 2.569, *p* = .01, bootstrap CI [0.071, 0.527]). Specifically, females are more likely to develop panic spells in adolescence compared to males, and the occurrence of panic spells increases for females if their mother went hungry in the first year of the adolescent's life (see Figure 2).

Maternal reports of mom going hungry in first year of child's life positively associated with adolescents receiving a diagnosis of depression or anxiety at 15 years of age (*r* = .382, *p* = .013). Primary caregiver reports of going hungry during child's ninth year positively associated with 15‐year‐old reported frequency of intentionally hurting another person and causing serious injury (*r* = .345, *p* = .025), and adolescent reported frequency of feeling restless and anxious (*r* = .353, *p* = .022).

### 3.2 Gene–environment interactions

**FIGURE 3:**
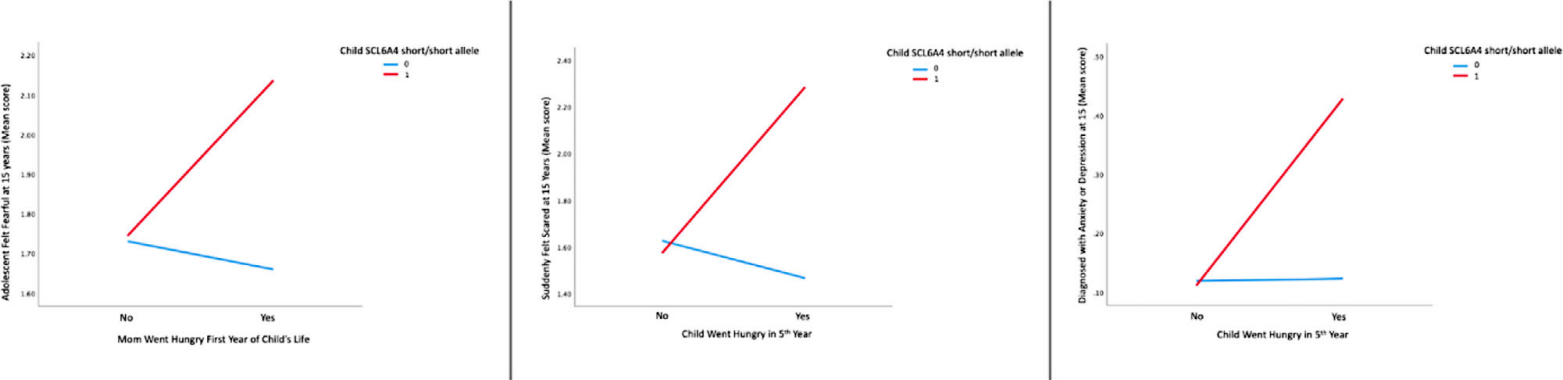
Gene X environment interaction with SCL6A4 short/short allele and food insecurity with adolescent anxiety features.

The SLC6A4 serotonin transporter short/short allele emerged statistically significant as a moderator of food insecurity and child mental health shown in Figure 3. Multiple regression indicated that adolescents with the short/short allele who had mothers who experienced going hungry in the adolescents first year of life were more likely to report a greater frequency of feeling fearful at 15 years compared to carriers of the long/long allele (*B* = 0.413, *t* = 1.972, *p* = .049, bootstrap CI [0.002, 0.823]). Adolescents with short/short allele who reportedly went hungry during their fifth year were more likely to report a greater frequency of unexpectedly suddenly feeling scared compared to carriers of the long/long allele (*b* = 0.718, *t* = 2.033, *p* = .042, bootstrap CI [0.026, 1.409]). Multinomial logistic regression comparing the short/short and long/long allele carrier groups revealed adolescents with the long/long allele who did not skip meals due to food insecurity during their fifth year were 478% less likely to be diagnosed with depression or anxiety at 15 years of age compared to peers with the short/short allele (*B* = 1.754, Odds ratio = 5.78, *p* = .047, bootstrap CI [1.026, 32.548]).

The SLC6A4 serotonin transporter long/long allele emerged statistically significant as a moderator of food insecurity and adolescent delinquent behavior. Multiple regression moderation analysis, of the interaction term and genetic and food insecurity variables as controls, indicated that mothers who reported going hungry in the child's first year of life had adolescents who reported a greater frequency of using or threatening to use a weapon to steal from a person at 15 years if they had the long/long allele in comparison to the short/short allele (*B* = 0.090, *t* = 2.744, *p* = .006, bootstrap CI [0.026, 0.154]). The long/long allele was not a statistically significant moderator of food insecurity for the child and adolescent mental health or other delinquent behaviors (*p* > .05), or of food insecurity for the mother or primary caregiver and adolescent mental health or other delinquent behaviors (*p* > .05).

### 3.3 Timing and chronicity of food insecurity

The present study employed analysis of covariance (ANCOVA) to further examine the temporal effects of food insecurity. Two summary variables were generated, one for the mother and one for the child, to indicate the duration and frequency of exposure to food insecurity. The food insecurity data for the mother, during the adolescents' childhood, were collected at three specific time points: year 1, year 5, and year 9. The variable indicating the timing of exposure (MFIT) was categorized into three groups: no exposure (coded as 0), early exposure (coded as 1, indicating exposure in year 1), late exposure (coded as 2, indicating exposure in either year 5 or year 9), and chronic exposure (coded as 3, indicating exposure at two or more time points). The available data for adolescents were limited to years 1 and 5 exclusively. The variable representing the timing of exposure to the adolescents (CFIT) was categorized into three groups: early, late, and chronic exposure. These categories were assigned numerical codes, with 0 indicating no exposure, 1 indicating early exposure in the first year, 2 indicating late exposure in the fifth year, and 3 indicating exposure at both time points. The study examined the timing of food insecurity effects on four outcome variables that were found to be statistically significant in gene–environment interactions.

The gene and maternal food insecurity interaction effects on adolescent self‐report of feeling fearful and threatening with a weapon to steal from another person were tested for food insecurity timing and chronicity effects (MFIT). The analysis of covariance (ANCOVA) findings revealed that MFIT did not exhibit a significant direct effect on feeling fearful among adolescents (*p* > .05). Consequently, no further evaluation of MFIT and feeling fearful was conducted. However, MFIT did demonstrate a statistically significant disparity in the average scores pertaining to the frequency at which adolescents engage in threatening with a weapon to steal from another individual. In particular, adolescents whose mothers reported skipping meals on two or more of the timed data collections exhibited a higher average propensity to engage in threatening with a weapon for theft, compared to adolescents whose mothers reported no instances of food insecurity (mean difference = 0.0500, SE = 0.01467, *p* = .004), and whose mother reported food insecurity only at year 5 or 9 (mean difference = 0.0552, SE = 0.01726, *p* = .008). There was no statistically significant difference in the means between the exposure to food insecurity at year 1 and multiple exposures (*p* > .05).

Given the significant results for MFIT and threatening with a weapon to steal, the gene–environment interaction was tested again with the new summary food insecurity variable for mothers (MFIT) to determine if an interaction was still present when accounting for timing and chronicity of exposure. ANCOVA results indicated that MFIT and the LL allele did not interact in association with the mean scores of adolescent threatening with a weapon to steal (*p* > .05), but MFIT continued to show a direct effect as previously noted (*p* = .016).

The adolescent food insecurity during childhood and gene interaction effects on adolescent self‐report of suddenly feeling scared and a clinical diagnosis of depression or anxiety were further tested for timing and chronic food insecurity (CFIT) effects. ANCOVA results indicated that CFIT did not have a direct effect on adolescent's suddenly feeling scared or a clinical diagnosis of depression or anxiety (*p* > .05), and therefore was not further examined.

## 4 DISCUSSION

The findings of this study emphasize that if both the mother and child experience food insecurity throughout the early years of life, they are likely to persist in facing hunger, even when provided with food resources. Furthermore, it is probable that the issue of food insecurity experienced during the initial year of a child's life will persist during the subsequent 10 years, and therefore likely explains the lack of difference in some of the outcome measures between early and chronic exposure to food insecurity. Adolescents with compromised psychological development exhibited a higher likelihood of having experienced maternal malnutrition throughout childhood. The frequency of panic periods was shown to be higher among these teenagers, particularly in females. In the context of food insecurity, it was shown that females exhibited a higher propensity for the development of anxiety disorders.

The discourse surrounding the issue of food security necessitates ongoing attention, even in the face of evolving social policies that aim to address this societal challenge. In 2014, a comparative analysis of 10 established Western nations revealed that the United States of America obtained the lowest overall ranking. Specifically, it rated fifth in terms of poverty and eighth in terms of economic mobility (Grusky et al., 2016). According to the World Economic Forum (2020) (WEF), the United States is positioned at the 27th rank in terms of social mobility, which is characterized as the capacity for offspring to experience improved living conditions compared to their parents. The WEF (2020) also reports that the United States lags below 26 other countries in terms of social mobility, namely, in the capacity for individuals to ascend the socioeconomic ladder. The prospects for upward mobility among individuals residing in poverty in the United States, as well as their offspring, are notably bleak. Policies should be designed to ensure fair and inclusive access to food resources, thereby enabling individuals experiencing severe poverty to fulfill their fundamental survival requirements without the burden of food insecurity for their families.

Regarding gene–environment interactions, it has been observed that the presence of the SLC6A4 serotonin transporter short/short allele plays a moderating role in the relationship between food insecurity and mental health among adolescents. Adolescents who had the short/short allele and whose mothers experienced nutritional deprivation during their initial year of life exhibited a higher propensity to self‐report experiencing fear. If a teenager experienced food deprivation throughout their fifth year of life, they had a higher likelihood of reporting unexpected feelings of panic. The findings indicate that adolescents who do not possess the short/short allele and do not experience food insufficiency throughout their fifth year of life exhibit a significantly reduced likelihood (478% less) of developing symptoms of sadness or anxiety. The results of our study align with prior research that suggests the long/long allele has a role in fostering resilience among children and adolescents (Niitsu et al., 2019). Nevertheless, recent research has revealed that teenagers possessing the long/long allele are prone to encountering mental health challenges resulting from stress, unless they exhibit high levels of resilience as assessed by the Connor‐Davidson Resilience scale (Ollmann et al., 2021).

The lack of consistency in the results pertaining to stress‐inducing experiences, mental health, and the SLC6A4 gene could be attributed to the unaccounted factor of an individual's psychological resistance to stress, which was not assessed in our study (Sharpley et al., 2017). This observation highlights the intricate nature of the gene–environment interaction model, implying the existence of additional influential components that contribute to an individual's psychological resilience, such as the shared family environment and coping mechanisms focused on emotions (Navrady et al., 2018). Food insecurity can arise from, as well as contribute to, parental mental health concerns that impact the overall family environment and the development of emotion control in offspring during their childhood and adolescence.

The inclusion of timing and chronicity of food insecurity as factors did uncover an impact on a particular gene–environment interaction related to delinquent behavior and the LL allele. This observation underscores the intricate nature of food insecurity and its methods of assessment. The consideration of interaction effects that arise from specific times of exposure is crucial within the broader framework of other timed exposures and chronic exposure. The findings of our study suggest that there are distinct impacts on child development when comparing early versus late food insecurity and late versus chronic food insecurity. These effects become evident in the mental health and delinquent behavior of adolescents.

To enhance the comprehensiveness of future investigations on the impacts of food insecurity, it would be advantageous to incorporate assessments of psychological resilience, the shared environment within families, and the regulation of emotions in children and adolescents. This would help elucidate the mechanisms through which gene–environment interactions, specifically concerning SLC6A4 gene variations, exert their influence. Similar to the transition toward precision medicine (Eid et al., 2019), the investigation of gene–environment interaction has the potential to contribute to policy creation and advocacy efforts aimed at enhancing developmental outcomes for children and adolescents. The objective of this study is not to ascertain “genetic risk” since the variants under investigation are prevalent among the general population. However, by enhancing our comprehension of how genetic variation in humans interacts with risk and protective experiential factors, we can obtain empirical evidence to bolster the case for federal legislation aimed at mitigating food insecurity in the United States.

It is important to take into account the constraints of this study when interpreting these results. Initially, it is important to note that food insecurity can be conceptualized as a stressor, but it is also imperative to acknowledge its potential nutritional implications, which can significantly impact human development and overall well‐being. The inclusion of nutritional components in our study was not feasible, hence limiting their potential contribution to our findings. Furthermore, our comprehensive model did not allow for the evaluation of the potential impacts of psychological resilience on stress. The relevance of psychological resilience in the route mechanisms of stress effects on mental health and behavior has been previously acknowledged. Nevertheless, this preliminary inquiry offers a fresh perspective on the issue of food insecurity and its detrimental impact on the mental well‐being and behavioral patterns of adolescents. These findings can serve as a basis for further exploration and contribute to the enhancement of the proposed theoretical framework. In order to overcome the limitations, we employed the sophisticated statistical technique of bootstrapping to mitigate the risk of type I error and derive population parameters for each model under investigation. This approach would decrease the probability of erroneous associations and provide additional evidence that the inclusion of recommended variables could augment, albeit not supplant, the food insecurity variable in the model.

## AUTHOR CONTRIBUTIONS

S. Pilkay and M. Nolasco developed the study. S. Pilkay conducted all analyses, wrote the methods and results, contributed to the introduction and discussion sections, and edited the manuscript. M. Nolasco contributed to the introduction and discussion sections, and edited the manuscript. S. Nunes, A. Riffer, D. Femia, D. Goldberger/Halevy, S. Heiland, N. Suwannimit, and N. Trexler verified results and interpretation, provided feedback and guidance on selection of mental health variables used in the study, and edited the manuscript. B. Gump provided analysis support, created a new construct variable for analysis, verified results and interpretation, provided feedback on study design, and edited the manuscript.

## FUNDING

No funding was received for conducting this study

## CONFLICT OF INTEREST STATEMENT

The authors declare that we have no conflicts of interest to report. The data used for this study are available through the Future of Families and Child Wellbeing Study at Princeton University.

### PEER REVIEW

The peer review history for this article is available at https://publons.com/publon/10.1002/brb3.3426.

## Data Availability

The data that support the findings of this study are available from the corresponding author upon reasonable request.
